# Organic ISFET Based on Poly (3-hexylthiophene)

**DOI:** 10.3390/s100302262

**Published:** 2010-03-19

**Authors:** Giuseppe Scarpa, Anna-Lena Idzko, Anandi Yadav, Stefan Thalhammer

**Affiliations:** 1 Institute for Nanoelectronics, Technische Universität München, Arcisstrasse 21, D-80333 Munich, Germany; E-Mail: anandi.yadav@mytum.de; 2 Helmholtz Zentrum München, German Research Center for Environmental Health, Institute of Radiation Protection, Ingolstädter Landstrasse 1, D-85764 Neuherberg, Germany; E-Mail: anna-lena.idzko@helmholtz-muenchen.de

**Keywords:** organic ISFET, P3HT, biosensing

## Abstract

We have fabricated organic field-effect transistors (OFETs) with regioregular poly(3-hexylthiophene) (P3HT) operable at low-voltages in liquid solutions, suitable for *in vitro* biosensing applications. Measurements in electrolytes have shown that the performance of the transistors did not deteriorate and they can be directly used as ion-sensitive transducers. Furthermore, more complex media have been tested, with the perspective of cell analysis. Degradation effects acting on the device operating in liquid could be partly compensated by adopting an alternate current measuring mode.

## 1. Introduction

Piet Bergveld introduced in 1970 the concept of an ion-sensitive solid-state device, based on a field-effect transistor, for electrophysiological measurements [1]. Afterwards, ion-sensitive field effect transistors (ISFETs) have found growing interest in the quickly evolving field of biosensors and have been implemented and customized in a variety of ways [2]. Their main advantages are fast response, high sensitivity, batch processing capability, microsize, solid-state structure, as well as the possibility of integrating output signal processing electronics forming a built-in integrated monolithic sensor system. Furthermore, ISFETs can be easily modified to enable sensing of certain biochemical substances, by the incorporation of, for example, enzymatic layers in enzyme-sensitive field-effect transistors (ENFETs), whole cell or biological layers in BioFETs or probe DNA nucleotides in DNA-FET-sensors [3]. Despite these considerable advantages, conventional ISFETs based on CMOS manufacturing processes suffer from problems related to long-term stability and fabrication costs. Field effect transistors based on organic materials (OTFTs) can provide a promising answer in that sense [4]. Beside low-cost large-area substrate-independent fabrication capability, they offer a unique opportunity when used in sensing application, in which the semiconducting material acts both as active transport layer and sensing component. In fact, both molecular structure and morphology can be adjusted and modified by specific receptor sites to fine-tune the chemical and physical properties and at the same time enhancing sensitivity and selectivity. In recent years, the use of OTFTs as sensors has steadily increased and several articles reviewing this field are available in the literature [4–6]. In the vapor phase, OTFTs can be easily operated as gas sensors. However, biological analytes and biologically derived recognition elements are only active in aqueous media. Therefore, it is necessary to explore OTFTs operation for aqueous systems, thus extending their applicability to a broader range of sensing needs. Although ion sensitive field-effect transistors [7–9] and sensors used in liquids [10–12] have been reported, the organic semiconducting layer is commonly not directly exposed to the electrolytes and the transduction mechanism is based on the charging effects occurring at the electrolyte / insulator interface. Someya *et al.* [13] were the first to explore OTFTs operation with the active semiconducting material in direct contact with both stationary and flowing water. By means of a special coating, they confined the water only to the active region of the transistor. Recently, stable operation in aqueous solution for a range of different organic semiconductors has been reported [14,15]. Low operating voltages and environmental stable organic materials are the key issues for devices operable in liquid solutions. Low driving voltages avoid unwanted electrochemical reactions as well as action potentials in case of cell signal studies. Regarding the materials, thermally evaporated organic semiconductors, which are stable in air, seem to be unaffected by the operation in water. On the other hand, solution-deposited semiconductors seem to suffer from water-induced degradation and/or delamination effects, eventually due to residual solvent at the dielectric interface [15]. However, solution-processable materials are a very attractive alternative for mass production of devices with large areas at low cost and deserve much more effort on the way to mature commercial applications. Previously, we designed and fabricated low-voltage operating organic thin-film transistor devices, which can be used as sensors in electrolytes [16]. The devices are based on regioregular poly (3-hexylthiophene) (P3HT), which is a reasonably conductive, optically active polymer with good environmental and thermal stability. Furthermore, we demonstrated the biocompatibility and biofunctionalization of the devices [17], which make them appealing also to understand complex cellular processes. Biological systems from cells to tissue are inherently dynamical systems, with responses to intracellular and extracellular inputs. Here, we describe operation of the devices in aqueous solutions and their sensitivity to different ions. The paper is organized as follows. Results are presented in Section 2, while Section 3 describes in detail the experimental set-up used for the measurement of the P3HT transistors.

## 2. Results and Discussion

First, it is important to understand the behavior of the transistors under various physical and environmental factors. The transistors are sensitive to changes in temperature, pH and ions in solutions. Moreover different complexes media, which are relevant for cell analysis, have been also evaluated. The following sections cover the characterizations in detail.

### 2.1. Measurement in Ambient Conditions and Thermal Behavior of Transistors in Liquid

**Figure 1.**
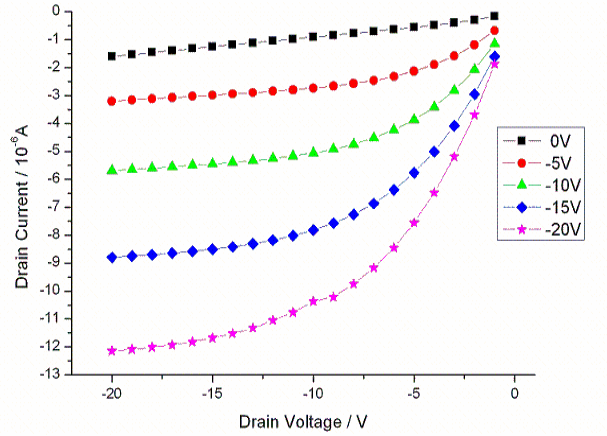
Output characteristics of a transistor with a 20 μm channel length after measurement in ambient conditions. The transistor shows a typical p-type characteristic.

Once the transistors were fabricated, their basic characterization was performed to ensure the proper working of the devices. The output characteristic of the transistors was measured at room temperature with the gate voltage being increased from 0 to −20 V in steps of −5 V, while the drain voltage was increased from 0 to −20 V in steps of −1 V. The sweep delay was kept at 1 s and the hold time before measuring the output characteristics was 1msec. Since the silicon dioxide was not treated separately, all transistors show a positive threshold voltage of about +5 V. Already at zero gate voltage, a clearly conducting channel could be seen which enables us to choose a low working point for current modulation measurements [16]. Figure 1 shows typical transistor characteristics at ambient condition.

The temperature sensitivity of the P3HT transistors in liquid was carried out by plugging the transistor set-up (with sample holder) into the PVC-box. The o-rings were filled with distilled water such that the devices and the temperature sensors were completely dipped in it [18]. The PVC-box was then kept in an oven for about two hours to heat the set-up to 37 °C, which is the optimum temperature for cell analysis experiments. Once heated, the transistors were connected to the measurement set-up and were characterized while cooling down the system. In the temperature range between ambient conditions up to ∼40 °C, the changes in transistor current nonlinearly varied with temperature, so that calibration curves would be necessary if one intends to analyze culture media in dynamic cell and tissue experiments.

### 2.2. Ion sensitivity of the Transistors

**Figure 2.**
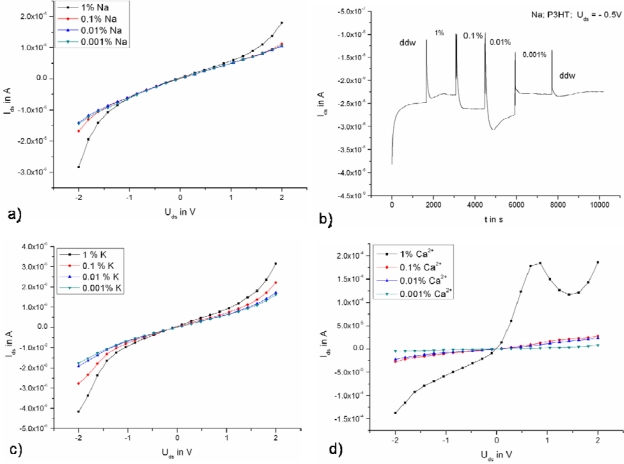
Output characteristics of a P3HT transistor (50 μm channel length) with different ion concentrations of **(a)** Na^+^, **(c)** K^+^ and **(d)** Ca^2+^. **(b)** Transistor current *versus* time, continuously monitored with varying Na^+^ ion concentration solutions.

For ion-sensitivity measurements, ion standard solutions (c (K/Na) = 1.000 ± 0.002 g/L, KCl/NaCl in water; c (Ca) = 1.000 ± 0.002 g/L; Ca (NO_3_)_2_ * 4H_2_O in HNO_3_ 0.5 mol/L; Merck) were used. The concentration of the ion solutions was varied from 1% to 0.001%. The measurements were performed in a flow chamber. Before every measurement, the transistors were rinsed with distilled water. Then the test solution was placed in the chamber and the measurements were performed. The applied drain-source voltage was varied from −2 to 2 V (with open gate) and the transistor current was measured simultaneously. It is worth mentioning that due to the field-effect enhanced transduction mechanisms on which the sensors rely, the devices show amplified sensitivity, which has already led to sub-ppm levels of detection [15,19,20]. Figure 2 (a), (c) and (d) show an overview of the obtained results for Na^+^, K^+^ and Ca^2+^ ions.

In the case of Figure 2 (b) the source-drain current was measured *versus* time with floating gate and a constant source-drain voltage of −0.5 V in the following way. A droplet (∼40 μl) of double-distilled water (ddw) was put first on top of the transistor using a pipette and the measurement was started. After the time required for stabilization, a droplet of ion solution was placed on top of the transistors after removing the ddw. This procedure was repeated each time the ion concentration was changed. This measurement demonstrates that online operation of the devices is feasible. It can be seen that the transistor is sensitive to the varying ion concentration. The change in the drain-source current is the highest with increasing Ca^2+^ concentration (probably due to the HNO_3_ present in the solution) ΔI_ds_ (Ca^2+^; U_ds_ = −2 V) = 1.4 × 10^−4^A, ΔI_ds_ (K^+^; U_ds_ = −2 V) = 2.2 × 10^−6^A, and ΔI_ds_ (Na^+^; U_ds_ = −2 V) = 1.3 × 10^−6^ A. It is important to note that at high ion concentrations with voltages higher than 1V, ionic current also contributes, as apparent by the feature in Figure 2 (d) at 1% Ca^2+^ ion concentration. The measurements were repeated many times and always showed the same results.

### 2.3. pH Dependence

**Figure 3.**
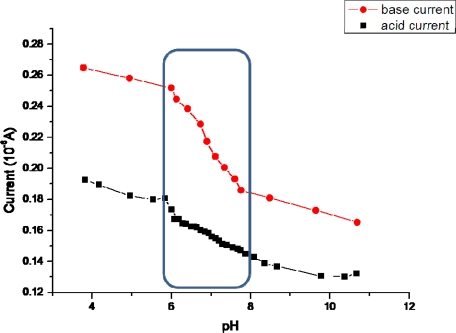
Transistor current behavior under various pH conditions. The transistor current was measured in DC mode with the drain-source voltage at 1 V and open gate. The enclosed area in the figure depicts the linear working range of the transistors from pH 6 to pH 8.

In order to analyze transistor behavior under various pH solutions, the following experiment was performed. Firstly, a buffer solution was prepared with 0.1 M of NaCl and 10 mM of HEPES dissolved in distilled water. HEPES was used in this experiment, since it is an organic buffering agent that is present in commonly used cell media to stabilize the pH of the tissue medium around pH 7. Separately, an acidic and a basic solution were made. The acidic solution was prepared by dissolving hydrochloric acid (HCl) in 200 ml of the buffer solution, while the basic solution was prepared by dissolving sodium hydroxide (NaOH) in 250 ml of the buffer solution. All solutions were heated-up and maintained at 37 °C for emulating optimum cell response conditions. Before starting the measurement, the buffer solution’s pH was adjusted to pH 4 by adding sodium hydroxide. At this point, the entire chip setup was immersed in the buffer solution and the other end was connected to the measuring units. The drain-source voltage was kept at 1 V (with open gate) and the transistor current was measured simultaneously with respect to pH changes of the solution. The pH was changed in very small steps from pH 4–10 by adding 0.5 mL of the acid solution to the buffer at each step and the current values were noted after a rough stabilization period of 10 min (acid current curve in Figure 3). At the end of the acid measurements (around pH 11), the basic solution was added (again in steps of 0.5 mL), thereby changing the pH values of the solution back from pH 10 to pH 4 (base current curve in Figure 3). The pH value was measured using a pH meter, type CyberScan 500 (Eutech Instruments Europe, Nijkerk, The Netherlands) and the results are shown in Figure 3.

The graph shows that within the cell working range (*i.e.*, in the pH range from 6–8, see marked area within the figure), the pH value changes were smaller than in the range beyond pH 8 and before pH 6 while adding the same amount of acid/base. This is due to the presence of HEPES in the buffer solution. Regarding the transistor response, we see that within the cell working range of the pH, the dependence of the transistor current with the pH is almost linear [21].

The current shift between the two types of measurements can be partly attributed to the different contribution of the ionic currents and/or changes in the polymeric layer due to the prolonged measurement time. The ionic current contribution can be compensated with an analog electronic interface, which amplifies only the pH-linear dependent component of the transistor current [21].

### 2.4. DC and AC Characterization

Typically the characterization measurements are performed in direct current (DC) mode. So far, the transistors were first tested in aqueous media in DC mode in order to see the reaction of the polymer when DC is applied to it. However, DC mode could be responsible for polarization effects, which in turn could damage the polymeric layers by degradation and/or delamination. Alternate current (AC) measuring mode could be interesting for preventing liquid-related damages and prolonging the lifetime of the devices. With the perspective of performing sensing experiments in complex media relevant for cell measurements, device characterization was done with fluid constituents of the RPMI cell culture media. Table 1 summarizes the experiments performed with our transistors. The time columns give the time for which the transistors were stable for a good measurement. The measurement time was varied from five min to 12 h. The signs ‘+++’, ‘++’, ‘+’, ‘−’, ‘−−’ and ‘−−−’ indicate the level of effects of the various solutions on the polymer from best (+++) to worst (−−−).

**Figure 4.**
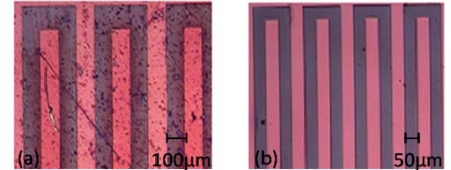
Optical microscopic images representative of the grading scale used in Table 1. (a) ‘worst effect’ (grading scale −−−) and (b) ‘no effect’ (grading scale +++).

**Figure 5.**
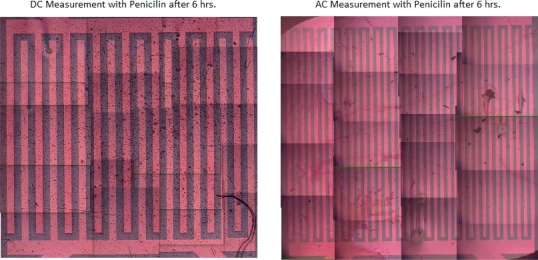
Comparison of DC and AC measurement modes with penicillin solution on top of the transistors after 6 h. The DC measurement resulted in deposits on the transistor degrading it, whereas the transistor was working after the similar measurement was repeated with AC.

The measurement with RPMI medium complete with 10% penicillin and FKS reacted with the polymer within five min after starting the measurement and degraded the transistor completely. Therefore, individual components of the RPMI medium were tested with the transistors. Indeed, penicillin and FKS also react with the transistors, forming either deposits or rivulets on the surface of the transistors. An AFM analysis of these transistors showed that there were deposits on the surface of the transistors instead of etching. For all these measurements, the images of the transistors were taken before and after each experiment. Some experiments with amino acids that are present in the RPMI medium were also performed, and it was noticed that the amino acids dissolved in ddw, did not react as much with the transistors as the ones dissolved in PBS. Other experiments were done only with PBS on the transistors, which showed no change in the functioning of the transistors before or after the measurement. Similarly, constituents like glucose (C_6_H_12_O_6_) and sodium hydrocarbonate (NaHCO_3_) had no effect on the polymer whatsoever. Since the penicillin and FKS were reacting badly with the polymer, they were excluded from the RPMI medium and the measurement was done again. This resulted in the transistors functioning properly even after six hours of measurement. Figure 4 shows two optical images after two hours DC characterization (a) with FKS solution on top and (b) with PBS solution on top. The two images are representative of the relative grading scale introduced in Table 1. Figure 5 shows the comparison between DC and AC measurements done with penicillin. Please note that in this case several images were assembled together to form the entire transistor structure, due to limitations of our set-up.

For all AC experiments, the frequency was kept at 1 Hertz. Some experiments were performed with the transistors coated with proteins such as collagen and fibronectin on top and kept in the incubator at 37 °C for about 20 min. After this, they were cleaned with PBS, dried and then the measurements were started with the RPMI medium on top of the transistors. For all the fluid measurements, approximately 40 μL of fluids was put on top of the transistor. Also the measurements with proteins were stable to quite an extent. One possible explanation for this could be that the collagen and fibronectin form a protective layer on top of the P3HT, preventing it from damage. The measurements with penicillin and FKS were repeated with AC and it was found that the penicillin did not react with the polymer under AC and the transistors worked fine, but the FKS reacted with the polymer again, degrading it within 15 min of the measurement. There was also some lightening of color at the electrodes. This was due to the bias applied at the transistor terminals which resulted in alternate light and dark patterns on the interdigitated electrodes. The P3HT transistors were tested under measurements carried out for long periods of time, approximately 12 hours, with RPMI medium on top of the transistors. Apparently the AC did not react that much with the polymer and the transistors worked fine even after 12 hours of measurement. The measurements were taken at 2, 4, 6 and 12 hours. There was some degradation and deposits on the transistor surface, but the transistor was tested for correct functioning after measurement and the overall functioning of the transistors was still good.

## 3. Experimental Section

The entire experimental set-up has been developed individually during the course of the research work and is briefly described here.

### 3.1. Sample Holder

Two kinds of sample holders, both made of PVC, were used for different kinds of experiments. One kind was used for the temperature sensing experiments, where two P3HT transistors were glued to a glass slide using silicone rubber for insulation and further wired to a sample holder with gold wires of diameter 0.1 mm with the help of silver glue for electrical connection. Coating the gold wires and silver glue with silicone rubber ensured an electrical passivation. Two rubber o-rings were placed around the passivated transistors and filled up with water for temperature sensing measurements. The other end of the gold wires was soldered to an electrical connector rail, which was further soldered to a miniature silicon temperature sensor type KTY 11-6 (Infineon, Neubiberg, Germany). The glass slide was mounted onto a PVC sample holder and wired with silver wires to a connector block. Teflon insulated gold connectors, type SMB EB2-L174 (Reichelt Elektronik, Sande, Germany) were used.

The other sample holder was without the temperature sensors, where the electrical rail was soldered to connecting wires, which were coupled together to one inner end of the PVC-box where the voltage was applied. The outer end of the box was connected to coaxial cables which were in turn connected to the semiconductor parameter analyzer. This set-up was used for all the measurements except the temperature sensing experiments.

### 3.2. Fabrication of P3HT-Based Transistors

The semiconducting regioregular-P3HT polymer was purchased from Sigma-Aldrich. The layout consists of a silicon substrate also acting as the gate of the transistor. It is coated with a gate oxide of silicon dioxide with a thickness of 45 nm. The transistor consists of an interdigitated pattern of electrodes acting as source and drain of the transistor. The area of each transistor is approximately 7 mm in size. The channel length of the transistors varies from 10–100 μm. Once the wafers are fabricated, the gold and titanium contacts were evaporated onto the transistors in a thermal evaporation chamber. The thickness of Ti is 2 nm and of Au used is 45 nm with a deposition rate of 1.0 Ǻ/s for Au and 0.2 Ǻ/s for Ti. Alternately, sputtering was also used for metal deposition. The P3HT solution was prepared with 0.06g of P3HT in 6 g of chloroform (1% weight) in a glass bottle with a magnet inside. The P3HT solution was put in an ultra sonic bath for 15 min, so as to dissolve the P3HT material completely in chloroform. The prepared P3HT solution was spin coated onto the surface of the transistor at 2000 rpm for 30 s. Once the spin coating is done the transistors are heated for about 10 min at 60 °C, so as to remove any toxic solvent residues. The wafer is then cut to separate each transistor using a diamond tip cutter.

### 3.3. Experimental Set-Up

**Figure 6.**
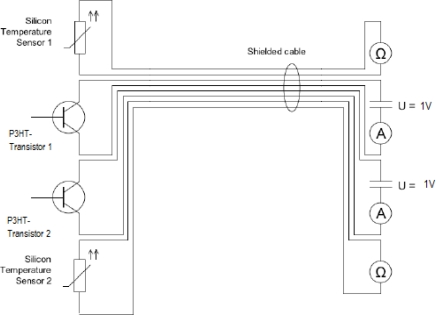
Schematic representation of wire set-up used for temperature sensing using two silicon temperature sensors and two P3HT transistors wired coaxially with the data acquisition system.

The P3HT transistors were mounted on a sample holder and are connected with gold wires, which have a diameter of 0.1 mm. Electrical connections are assembled with shielded coaxial cables RG-174, gold connector plugs type SMB ST-C174, gold cable couplings type SMB KU-KU and gold connectors, type SMB EB2-L174 (Reichelt Elektronik, Germany). Data acquisition is accomplished with Keithley Source and Multimeters (Keithley Instruments Inc., Cleveland, Ohio 44139). A P3HT transistor is connected to a Keithley 2400 Source meter, which provides the possibility to apply a voltage and measure current simultaneously. Hence, a constant voltage of 1 V was applied to the source-drain contacts of the P3HT transistors. Silicone temperature sensors, type KTY 11-6 (Infineon, Neubiberg, Germany), were connected to a Keithley 2000 and a Keithley 2700 multi-meter for the acquisition of the temperature. The Keithley devices send their data to a GBIP Interface Board, type KPCI-488LP IEEE-488.2 (Keithley Instruments Inc., Cleveland, Ohio, 44139), which is installed in a Windows XP based PC. Control and read-out of the measurement devices, as well as storage of the sampled data, was done with Labview 8.6 Express (National Instruments Dtl., Muenchen, Germany). Figure 6 depicts the schematics of the experimental set-up used.

## 4. Conclusions

In this report, we discussed the stability of P3HT-based OTFTs and their use in electrolytes and more complex media, which will play an important role for realistic biological detection needs. We showed the influence of water and ions on the device characteristics. In our organic ISFET the semiconducting material is in direct contact with the analyzing solution. Ions and analytes can diffuse through the polymer reaching the dielectric interface, where the electrical conduction takes place. The influence of those molecules on the charge transport can then be detected. Although this mechanism is not fully understood yet, it can be exploited in the realization of functioning sensing systems working in aqueous environment. Problems related to degradation and delamination effects in water can be partly avoided by using AC measurement mode. Real-time detection of biological analytes can also be feasible and disposable biochemical sensors are definitely a viable application for these types of devices. This however still deserves a much higher research effort.

## Figures and Tables

**Table 1.** DC, AC measurement summary. The time columns gives the time for which the transistors were stable. The effect on the polymer under DC and AC is explained by relative grading ranging from +++ being ‘no effect’ to −−− being ‘worst effect’.

**Solutions**	**AC / Time**	**DC / Time**
Medium Complete	−− / 15 min	−−− / 5 min
Medium with Collagen	+ / 30 min	
Medium with Fibronectin	+ / 30 min	
PBS		+++ / 2 h
Glucose		+++ / 12 h
Penicillin	++ / 6 h	−− / 4 h
FKS	−− / 15 min	−− / 15 min
Medium w/o Pen/FKS		++ / 6 h
L-Arginine with ddw		+++ / 1 h
L-Arginine with PBS		−− / 15 min
L-Glutamin with ddw		+ / 4 h
L-Glutamin with PBS		−− / 30 min
NaHCO3		+++ / 6 h
Medium-long term	++ / 12 h	

## References

[1] Bergveld P. (1970). Development of an Ion-Sensitive Solid-State Device for Neurophysical Measurements. IEEE Trans. Biomed. Eng.

[2] Bergveld P. (2003). Thirty years of ISFETOLOGY: What happened in the past 30 years and what may happen in the next 30 years. Sens. Actuat. B.

[3] Yuqing M., Jianguo G., Jianrong C. (2003). Ion sensitive field effect transducer-based biosensors. Biotech. Adv.

[4] Bartic C., Borghs G. (2006). Organic thin-film transistors as transducers for (bio) analytical applications. Anal. Bioanal. Chem.

[5] Janata J., Josowicz M. (2003). Conducting polymers in electronic chemical sensors. Nat. Mater.

[6] Mabeck J.T., Malliaras G.G. (2006). Chemical and biological sensors based on organic thin-film transistors. Anal. Bioanal. Chem.

[7] Bartic C., Campitelli A., Borghs G. (2003). Field-effect detection of chemical species with hybrid organic/inorganic transistors. Appl. Phys. Lett.

[8] Loi A., Manunza I., Bonfiglio A. (2005). Flexible, organic, ion-sensitive field-effect transistor. Appl. Phys. Lett.

[9] Ji T., Rai P., Jung S., Varadan V.K. (2008). In-vitro evaluation of flexible pH and potassium ion-sensitive organic field effect transistor sensors. Appl. Phys. Lett.

[10] Sargent A., Loi T., Gal S., Sadik O.A. (1999). The electrochemistry of antibody-modified conducting polymer electrodes. J. Electroanal. Chem.

[11] Sargent A., Sadik O.A. (1999). Monitoring antibody-antigen reactions at conducting polymer-based immunosensors using impedance spectroscopy. Electrochim. Acta.

[12] Meijerink M.G.H., Strike D.J., de Rooij N.F., Koudelka-Hep M. (2000). Reproducible fabrication of an array of gas-sensitive chemo-resistors with commercially available polyaniline. Sens. Actuat. B: Chem.

[13] Someya T., Dodabalapur A., Gelperin A., Katz H.E., Bao Z. (2002). Integration and response of organic electronics with aqueous microfluidics. Langmuir.

[14] Roberts M.E., Mannsfeld S.C.B., Queralto N., Reese C., Locklin J., Knoll W., Bao Z. (2008). Water-Stable organic transistors and their application in chemical and biological sensors. Proc. Natl. Acad. Sci. USA.

[15] Roberts M.E., Mannsfeld S.C.B., Stoltenberg R.M., Bao Z. (2009). Flexible plastic transistor-based chemical sensors. Org. Elect.

[16] Goetz S.M., Erlen C.M., Grothe H., Wolf B., Lugli P., Scarpa G. (2009). Organic field effect transistors for biosensing applications. Org. Elect.

[17] Scarpa G., Idzko A.L., Goetz S.M., Thalhammer S. Biocompatibility studies of functionalized regioregular poly(3-hexylthiophene) layers for sensing applications. Macromol. Biosci.

[18] Hamadani B.H., Natelson D. (2004). Temperature dependent contact resistances in high-quality polymer field-effect transistors. Appl. Phys. Lett.

[19] Tanese M.C., Fine D., Dodabalapur A., Torsi L. (2005). Interface and gate bias dependence responses of sensing organic thin-film transistors. Biosens. Bioelectron.

[20] Torsi L., Farinola G.M., Marinelli F., Tanese M.C., Omar O.H., Valli L., Babudri F., Palmisano F., Zambonin P.G., Naso F. (2008). A sensitivity-enhanced field-effect chiral sensor. Nature Mater.

[21] Bartic C., Palan B., Campitelli A., Borghs G. (2002). Monitoring pH with organic-based field-effect transistors. Sens. Actuat. B.

